# EyeSustain: Pioneering specialty-specific sustainability

**DOI:** 10.1016/j.joclim.2025.100631

**Published:** 2026-02-12

**Authors:** Sruti Rachapudi, Sarishka Desai, Amy Song, Barbara Erny

**Affiliations:** a Illinois Eye and Ear Infirmary/University of Illinois Chicago, Chicago, IL, USA; b University of Connecticut School of Medicine, Farmington, CT USA; c University of Illinois College of Medicine, Chicago, IL USA; d Department of Medicine, Stanford University, Palo Alto, CA USA

**Keywords:** Sustainability, Ophthalmology, Climate change

## Abstract

Introduction: As physician awareness of our contributions to climate change increases, efforts to implement sustainable practices in ophthalmology are gaining momentum. Healthcare professionals are trusted voices in their communities and are therefore well suited to advocate for the systemic and policy changes necessary to address climate change and protect human health in equitable ways. Purpose: In this case report, we highlight the efforts of one physician to drive change and advocate for more sustainable practices in ophthalmology as well as the creation of EyeSustain, a global organization dedicated to this mission. Case Discussion: EyeSustain is a multisociety-backed, ophthalmology-specific sustainability initiative that centralizes resources, research, and information on sustainable practices in healthcare. The initiative has grown to include over 50 global member societies and is sponsored by major ophthalmology organizations, demonstrating widespread support for reducing waste and improving sustainability in ophthalmic practice. The efforts of one passionate physician, now an EyeSustain Advisory board member, are showcased and expanded upon on the website EyeSustain.org. EyeSustain.org serves as a comprehensive platform, offering tools, recommendations, and resources to help ophthalmologists and support staff reduce waste in operating rooms and clinics, develop sustainable practices, and to provide education about the ocular effects of climate change. As a centralized global resource, EyeSustain builds collaboration and provides clinicians and staff with tools and streamlined avenues for communication to foster a more sustainable healthcare system. Conclusion: The model of EyeSustain provides a blueprint for other medical specialties to follow in addressing health impacts of climate change, medical waste, and healthcare sustainability.

## 1 Introduction

Climate change, described by the World Health Organization as “the greatest threat to global health,” is already impacting populations worldwide and will only worsen in the future [1]. Healthcare contributes approximately 4.5% of global, and 10% of U.S. greenhouse gas emissions, with operating rooms accounting for about 70% of this waste [2]. Ophthalmology generates substantial medical waste through high-volume procedures such as cataract surgery and retinal injections [3,4]. A 2019 study found that nearly two-thirds of topical drugs were discarded after cataract surgery, leading to an estimated $195,000 wasted annually per surgery site on unused medication [5]. Cataract surgery alone accounts for $3.5 billion of the Medicare budget, yet much of the material with these surgeries is discarded, creating both significant financial and environmental strain [6]. With the number of worldwide cataract operations projected to rise from 29 million in 2019 to 50 million by 2050, the field’s environmental impact will only grow [7].

As awareness of the environmental strain of practice increases, efforts to implement sustainable practices in ophthalmology are gaining momentum. Urgent action is needed to address climate threats, promote greener systems, and communicate effectively about these issues.

### 1.2 Case report

In 2019, a Chicago-based ophthalmologist took note of the unnecessary drug waste created by the disposal of topical medications used in cataract surgery, as well as the financial burden created by requiring patients to purchase these same medications for post-op use. When established safety protocols are followed, the use of multidose ophthalmic solutions is considered safe. Since 1964, the U.S. Food and Drug Administration (FDA) has authorized multidose packaging of topical medications containing antimicrobial preservatives, provided they are labeled with an expiration date [7]. Multiple large-scale studies have demonstrated no associated increase in endophthalmitis risk with proper reuse of eye medication bottles on multiple patients [8,9]. In 2015, the American Society of Cataract and Refractive Surgery (ASCRS) issued a position statement reaffirming the practice of multidosing eye drops [10].

In his home state of Illinois, the ophthalmologist found laws and institutional policies often created a barrier for patients to take home intraoperative drops after a colleague at a different institution was sanctioned for sending opened eye drops used intraoperatively home with the same patient.

In response to these challenges to patient care, the ophthalmologist decided to take action to combat needless medical waste. Appalled that intraoperative topical eye drops were purchased and used for just one patient and then the entire bottle discarded, he drafted and submitted a resolution to the Chicago and Illinois State Medical Societies advocating for patients to take home their medications, when properly stored and labeled, for postoperative use. Once adopted, a modified resolution was introduced into the Illinois General Assembly and became a 2021 state law (PA 102–0155). Throughout the legislative process, collaboration with key stakeholders—including the Illinois Council of Health-System Pharmacists and the Illinois Health and Hospital Association—was essential in refining the language and ensuring safety standards. Multi-specialty support also ensued, with state societies in ophthalmology, dermatology, plastic surgery, otolaryngology**,** and emergency medicine expressing support for the passage of this law. While formal environmental impact data following the law’s adoption are still emerging, continued monitoring and data collection are underway to evaluate the policy’s broader clinical and environmental outcomes.

Since the passage of PA 102–0155, the American Academy of Ophthalmology (AAO) has created a legislative template based on the Illinois statute to support ophthalmologists and other advocates in pursuing similar policies across the country [11]. This template has been successfully utilized as an example to pass similar topical drug waste reducing legislation the Delaware, Tennessee, Nebraska, and Colorado state legislatures to date. In 2021, the American Medical Association (AMA) ultimately adopted a similar policy endorsing the safe use of multidose eye drops on multiple patients [12]. The support of the AAO and AMA ultimately resulted in a multi-eye society position paper on multidosing topical medication co-authored by several ophthalmic professional societies, further legitimizing the practice and offering guidance for safe implementation [13].

Beyond topical eye drop waste, this physician’s impactful advocacy helped to create EyeSustain, a global initiative advocating for sustainability in ophthalmology. Through EyeSustain, he and his advisory and editorial board colleagues educate the ophthalmology community with evidence-based resources on sustainable practices, advocacy, waste-reducing efforts, and relevant position papers while stimulating more sustainability research.

### 1.3 Discussion

#### 1.3.1 EyeSustain: the beginning

EyeSustain, launched on Earth Day 2022, is a multisociety-backed, specialty-specific sustainability initiative for ophthalmology [14]. Initially centralizing dispersed healthcare sustainability resources, it has grown to include original research, industry initiatives, climate and ocular health information, and insights from low and middle-income countries.

The initiative was founded and originally sponsored by the American Society of Cataract and Refractive Surgery (ASCRS) and the European Society of Cataract and Refractive Surgeons (ESCRS). The American Academy of Ophthalmology (AAO), the largest eye society in the world, and the European Society of Retinal Specialists have since joined as co-sponsors. Over 50 ophthalmologic societies worldwide are now members, committing to support the goals of reducing waste and making ophthalmology more sustainable (Table 1).

**Table 1:** EyeSustain Sponsoring Organizations and Global Member Societies.

· American Academy of Ophthalmology - Co-Sponsor · American Society of Cataract and Refractive Surgery - Co-Sponsor · European Society Of Cataract & Refractive Surgeons - Co-Sponsor · African Ophthalmology Council (AOC) · All India Ophthalmology Society (AIOS) · American Association for Pediatric Ophthalmology and Strabismus (AAPOS) · American-European Congress of Ophthalmic Surgery (AECOS) · American Glaucoma Society (AGS) · American Society of Retina Specialists (ASRS) · Argentine Council of Ophthalmology (CAO) · Asia Pacific Academy of Ophthalmology (APAO) · Asia Pacific Society of Cataract and Refractive Surgery (APACRS) · Asia-Pacific Vitreo-Retina Society (APVRS) · Association for Research in Vision and Ophthalmology (ARVO) · Australian Society of Cataract and Refractive Surgery (AUSCRS) · Austrian Ophthalmology Society (ÖOG) · Belgian Ophthalmic Syndicate (SOOS) · Brazilian Council of Ophthalmology (CBO) · Brazilian Society of Cataract and Refractive Surgery (BRASCRS) · Canadian Ophthalmological Society (COS) · College of Ophthalmology of Eastern, Central, and Southern Africa (COECSA) · Chinese Ophthalmology Society (COS) - collaborating society · Deutsche Ophthalmologische Gesellschaft (DOG) · European Association for Vision and Eye Research (EVER) · European Dry Eye Society (EuDES) · European Glaucoma Society (EGS) · European Society of Ophthalmology (SOE) · European Society of Ophthalmic Plastic and Reconstructive Surgery (ESOPRS) · European Society of Retina Specialists (EURETINA) · Finnish Society of Cataract and Refractive Surgery (FSCRS) · French Society of Cataract and Refractive Surgery (SAFIR) · German Society of Cataract and Refractive Surgery (GSCRS) · Hellenic Society of Cataract and Refractive Surgery (HSIOIRS) · Hungarian Cataract and Refractive Surgical Society (SHIOL) · Intraocular Implant and Refractive Surgery, India (IIRSI) · Japanese Ophthalmology Society (JOS) · Latin American Society of Cataract and Refractive Surgeons (ALACCSA-R) · Malaysian Ophthalmological Society (MSO) · Netherlands Ophthalmological Society (NOG) · Ophthalmological Society of South Africa (OSSA) · Outpatient Ophthalmic Surgery Society (OOSS) · ORBIS International · Pan-American Association of Ophthalmology (PAAO) · Portuguese Society of Ophthalmology (SPO) · Royal Australian and New Zealand College of Ophthalmologists (RANZCO) · Spanish Society of Implant-Refractive Eye Surgery (SECOIR) · Turkish Ophthalmology Association · United Kingdom and Ireland Society of Cataract and Refractive Surgery (UKISCRS)

#### 1.3.2 Structure and function

EyeSustain's structure consists of an advisory board of experienced ophthalmologists and sustainability experts, an editorial board of young ophthalmologists, a medical student committee with over 70 members, a global council addressing concerns of low- and middle-income countries (LMICs), and an industry advisory board. Particularly, the global council offers crucial insight into how excellent surgical care is provided in low resource settings even with the extensive reuse of supplies, achieving efficacy and safety outcomes comparable to high-income countries while reducing carbon footprint over 90 % [15]. Individuals contribute new information, which is first vetted and summarized, to the website as well as for distribution on social media. Paid staff support the website and administrative tasks.

The EyeSustain.org website covers six topics: reduction of OR and clinic waste, ocular health, sustainability efforts in LMICs, industry initiatives, and drug waste and advocacy. It hosts a library of over 80 relevant articles pertaining to these topics allowing users to quickly access resources, highlight research, share events, and network.

#### 1.3.3 Tools and achievements

EyeSustain provides ophthalmology-specific tools and recommendations to reduce waste, including an online carbon footprint calculator [16]. Surgical facilities can take a pledge to reduce waste and be credited on the website. MyGreenDoctor.org provides ophthalmology-specific advice for the reduction of clinic waste, as well as a discount for EyeSustain society members. Collaborating with industry partners, EyeSustain has achieved significant milestones, such as replacing paper instructions for use in intraocular lens boxes with QR codes, eliminating thousands of reams of paper annually [17]. This and many of EyeSustain's sustainability recommendations have been published as position papers endorsed by the AAO and ophthalmology societies from all 50 US states.

#### 1.3.4 Enhancing the work of task forces

As of 2022, only 12 of 111 U.S. medical society organizations (11 %) included climate change committees or task forces [18]. These task forces share a common objective of promoting education on climate health and sustainability within their respective fields, but there is considerable variation in the strategies used by these organizations to address climate change. For example, the American Academy of Pediatricians Council of Environmental Health is focused more on the nuances of identifying, preventing and treating pediatric environmental health problems, such as those caused by impurities in drinking water, exposure to indoor and outdoor pollution, and chemicals in food and personal care products [19].

While the ocular effects of climate change have been well described in literature, the initial and continuing concerns of EyeSustain include all aspects of human and planetary health that are affected by plastic pollution and climate change. To our knowledge, most other specialties do not have a dedicated repository of resources and education about climate change and health and sustainable specialty-specific practices. In 2024, the medical subspecialty of rheumatology developed an initiative to inform patients and healthcare professionals about the potential impact of environmental changes on rheumatic diseases (REACTRheum), which includes a subsection on practice management resources for outpatient rheumatology practices [20]. EyeSustain.org uniquely focuses on providing actionable specialty-specific sustainability resources, as well as providing detailed checklists, toolkits, and pledges which empower physicians, staff and institutions to be better stewards of the environment by reducing waste as well as costs.

### 1.4 Conclusion

The tireless efforts of one physician have not only generated law and policy changes, but inspired other ophthalmologists and trainees to pursue research and be advocates for sustainable healthcare. EyeSustain is an example of how a few passionate stakeholders built a global initiative centered around promoting sustainability in one medical subspecialty which has grown to include over 50 major eye societies worldwide. EyeSustain is not only educating eye care professionals, residents and medical students to reduce waste in their practices but also driving policy change and industry shifts. However, widespread adoption of these practices continues to face significant barriers such as limited funding, variable institutional buy-in, and the resistance to disrupting existing infrastructures. The EyeSustain initiative and website have examples for implementing and sharing sustainability measures on a global scale, providing a new blueprint for other medical subspecialties to follow so they may tangibly address the growing carbon footprint of healthcare.

## CRediT authorship contribution statement

**Sruti Rachapudi:** Writing – review & editing, Writing – original draft, Supervision, Conceptualization. **Sarishka Desai:** Writing – review & editing, Writing – original draft, Conceptualization. **Amy Song:** Writing – review & editing, Writing – original draft, Conceptualization. **Barbara Erny:** Writing – review & editing, Writing – original draft, Supervision, Project administration, Conceptualization.

## Declaration of competing interest

The authors declare that they have no known competing financial interests or personal relationships that could have appeared to influence the work reported in this paper.The author is an Editorial Board Member/Editor-in-Chief/Associate Editor/Guest Editor for this journal and was not involved in the editorial review or the decision to publish this article.
